# ELK4 Promotes Vasculogenic Mimicry in Oral Squamous Cell Carcinoma via Driving *DHFR* Transcriptional Activation

**DOI:** 10.32604/or.2025.069612

**Published:** 2025-12-30

**Authors:** Yongle Qiu, Kunshan Li, Wenjing Wang, Wenjuan Zhang, Jilun Liu, Yang Bai, Fei Xu, Jie Guo

**Affiliations:** 1 Department of Stomatology, The Fourth Hospital of Hebei Medical University, Shijiazhuang, 050000, China; 2 Department of Oral Surgery, The Second Hospital of Hebei Medical University, Shijiazhuang, 050000, China; 3 Department of Stomatology, Qinhuangdao Haigang Hospital, Qinhuangdao, 066000, China

**Keywords:** Oral squamous cell carcinoma, vasculogenic mimicry, metastasis, dihydrofolate reductase, ETS transcription factor ELK4

## Abstract

**Background:**

The regulatory mechanisms governing vasculogenic mimicry (VM) in oral squamous cell carcinoma (OSCC) remain largely undefined. This study aimed to identify critical factors and elucidate the epigenetic mechanisms underlying VM in OSCC.

**Methods:**

Bioinformatics analysis was performed utilizing single-cell RNA-seq, bulk RNA-seq, and histone H3 lysine 27 acetylation (H3K27ac) Chromatin Immunoprecipitation (ChIP)-seq data obtained from The Cancer Genome Atlas (TCGA) and Gene Expression Omnibus (GEO) databases. ChIP-qPCR was used to validate the binding of ETS transcription factor ELK4 (ELK4) to the dihydrofolate reductase (DHFR) enhancer. *In vitro* VM formation and invasion of OSCC cells were assessed using Matrigel-based tube formation and Transwell assays, respectively.

**Results:**

Elevated expression of VM-related genes predicts unfavorable prognosis in OSCC patients. High-dimensional weighted gene co-expression network analysis (hdWGCNA) identified epithelial subcluster C4 as most strongly associated with VM and metastasis. Three co-expression modules within this subcluster exhibited significant positive correlations with both phenotypic traits. Among the 30 eigengenes from the three modules, *DHFR* emerged as a key regulator of VM and metastasis. Knockdown or inhibition of DHFR significantly suppressed VM formation and invasion in OSCC cells. Mechanistically, ELK4 activated *DHFR* transcription through direct binding to its enhancer. DHFR overexpression rescued VM and invasion impairment induced by ELK4 knockdown.

**Conclusion:**

*DHFR* was a pivotal enhancer-regulated gene driving VM and metastasis in OSCC. ELK4 directly binds to *DHFR* enhancer regions to activate its transcription, thereby promoting these malignant phenotypes. These findings identified the ELK4/DHFR axis as a promising therapeutic target for anti-angiogenic intervention in OSCC.

## 1 Introduction

Oral squamous cell carcinoma (OSCC) is the most common malignancy of head and neck cancer [1]. Metastasis is the leading cause of poor prognosis in OSCC patients, posing a major therapeutic challenge in clinical management [2,3]. Comprehensive elucidation of the metastatic mechanisms is pivotal for the development of the effective targeted therapies.

Vasculogenic mimicry (VM) is an important model of intra-tumor microcirculation in which tumor cells form vascular-like structures independent of endothelial cells [4]. The VM-formed vascular-like networks not only meet tumor metabolic demands, but also facilitate metastasis [5–8]. Emerging evidence demonstrates that VM contributes to resistance against conventional anti-angiogenic therapies [9,10]. However, the molecular mechanisms regulating VM in OSCC remain poorly defined. Elucidating these regulatory mechanisms is essential for developing novel anti-angiogenic therapeutic approaches in OSCC treatment.

Enhancers are critical regulatory elements that drive transcriptional reprogramming [11,12]. The interaction between tissue-specific transcription factors and histone modifications at enhancer regions underpins the spatiotemporal control of enhancer activity [13]. Histone H3 lysine 27 acetylation (H3K27ac) is a well-established hallmark of active enhancers [14–16]. Aberrant enhancer function has emerged as a key molecular mechanism in cancer progression [17]. Comprehensive investigation of the collaborative regulatory network between enhancers and transcription factors is crucial for understanding the epigenetic mechanisms governing oncogenic progression.

The purpose of this study was to elucidate key regulators and the underlying mechanisms of VM in OSCC. The findings of this study reveal a potential novel therapeutic target to complement current anti-angiogenic treatments for OSCC.

## 2 Materials and Methods

### 2.1 Acquisition and Analysis of Bulk RNA-Seq Data

We obtained bulk RNA-seq data and accompanying clinical annotations from The Cancer Genome Atlas (TCGA; https://portal.gdc.cancer.gov) and transformed expression values to log2(TPM + 1). To construct an OSCC-specific cohort, we restricted cases to primary tumors arising in the oral cavity based on clinical metadata, including the tongue, buccal mucosa, floor of mouth, hard palate, alveolar ridge, and lip; samples from other head and neck sites (e.g., larynx or pharynx) were excluded. We further retained only TCGA sample types “Primary Solid Tumor” (code 01) and “Solid Tissue Normal” (code 11). Samples lacking RNA-seq data or required clinical information were removed. The final dataset comprised 322 OSCC tumor samples and 44 adjacent normal tissues, designated the TCGA-OSCC cohort.

The R package “ConcensusClusterPlus” (version 1.72.0) [18] was used to identify molecular subtypes of OSCC. Similarity distance between samples was assessed using the Euclidean distance metric. The K-means clustering algorithm was applied for consistent clustering (maximum clusters = 6, iterations = 100), with each iteration randomly extracting 80% of the total tumor samples. The optimal k-value for the consensus matrix was determined by evaluating the cumulative distribution function (CDF) curves and the area under the CDF curves. A clustering heatmap was plotted using the R package “pheatmap” (version 1.0.13). Associations between gene expression levels were assessed using Spearman’s correlation, with statistical significance defined as *p* < 0.01 and |*r*| > 0.2.

### 2.2 Acquisition and Analysis of Single-Cell RNA-Sequencing (scRNA-Seq) Data

scRNA-seq data from OSCC tissues were sourced from the Gene Expression Omnibus (GEO) database (https://www.ncbi.nlm.nih.gov/geo/, accessed on 29 October 2025) under accession number GSE181919. We included only scRNA-seq libraries annotated as primary oral cavity tumors, excluding those from other head and neck sites such as the oropharynx and hypopharynx.

scRNA-seq data were analyzed using the “Seurat” (version 5.3.0) R package [19]. High-quality sequencing data were included according to the following thresholds: 300–6000 detected genes and <15% mitochondrial genes. Batch correction was performed using “Harmony” (version 1.2.3) [20]. The top 3000 highly variable genes were identified using the “FindVariableFeatures” function. Principal component analysis (PCA) was performed using the “RunPCA” function to define the top 20 principal components. Cell clusters were identified using the “FindClusters” function with a resolution of 1.0. Uniform manifold approximation and projection (UMAP) was performed for nonlinear dimensionality reduction using the “RunUMAP” function. Cell types were manually annotated based on the expression of classical marker genes. Cell proportions were visualized using R package “ggplot2” (version 3.5.2). DHFR expression across epithelial subclusters was analyzed using the “FindAllMarkers” function.

### 2.3 High-Dimensional Weighted Gene Co-Expression Network Analysis (hdWGCNA)

hdWGCNA was performed on scRNA-seq data from the GSE181919 dataset using the “hdWGCNA” package (version 0.4.05) [21]. Briefly, a Seurat object was generated using the “SetupForWGCNA” function with a fraction of 0.05. Metacells were constructed via the “MetacellsByGroups” function using the K-nearest neighbors (KNN) algorithm. The expression matrix was established using the “SetDatExpr” function. Optimal soft threshold was determined using the “TestSoftPowers” function by maximizing the scale-free topology model fit while minimizing connectivity. A co-expression network was constructed using the “ConstructNetwork” function according to the identified optimal soft threshold. This process involved hierarchical clustering of genes based on the Topological Overlap Matrix (TOM) dissimilarity (1-TOM) using the “hclust” function with the average linkage method. The resulting gene dendrogram was pruned using a dynamic tree-cutting algorithm to identify distinct co-expression modules. Module eigengenes were derived using the “ModuleEigengenes” function. Intramodular connectivity (kME) was calculated using the “ModuleConnectivity” function. Spearman’s correlation was used to assess associations between module eigengenes and phenotypic traits.

### 2.4 Prognostic Analysis

Recurrence-free survival (RFS) analysis in OSCC patients was performed using the Kaplan-Meier Plotter platform (https://kmplot.com/analysis/) (accessed on 30 October 2025) with auto-selected best cutoff and 60-month maximum follow-up.

### 2.5 Transcription Factors Prediction

Transcription factor binding affinities to *DHFR* enhancer regions were quantified using the Transcription Factor Affinity Prediction (TRAP) method. Predictions were made against the JASPAR vertebrates database (https://jaspar.elixir.no/) (accessed on 30 October 2025), with human promoters serving as the control set, and *p*-values were adjusted using the Benjamini-Hochberg method [22].

### 2.6 Acquisition and Analysis of Chromatin Immunoprecipitation (ChIP)-Seq Data

H3K27ac ChIP-seq data for primary OSCC cells (HN120-Pri) and metastatic OSCC cells (HN120-Met) were obtained from the GEO database under accession number GSE120634. Raw sequencing reads were aligned to the hg19 reference genome using “Bowtie2” (version 2.5.4). H3K27ac peaks were called using the “findPeaks” function in the HOMER algorithm (version 5.1) with default parameters. H3K27ac signaling around the *DHFR* locus was visualized using the Integrative Genomics Viewer (IGV; https://igv.org; version 2.19.6).

### 2.7 Acquisition of Immunohistochemistry Data

The immunohistochemistry data for DHFR protein expression in OSCC and normal control tissues were obtained from The Human Protein Atlas (HPA) database (https://www.proteinatlas.org/).

### 2.8 Cell Culture

Human OSCC cell lines were purchased from the Japanese Collection of Research Bioresources Cell Bank (JCRB; Osaka, Japan): non-metastatic HSC-2 (Cat#JCRB0622), and metastatic HSC-3 (Cat#JCRB0623) and OSC-20 (Cat#JCRB0197). All cell lines were authenticated using short tandem repeat (STR) profiling and confirmed mycoplasma-free. Cells were cultured in Dulbecco’s Modified Eagle Medium (DMEM; Gibco, Grand Island, NY, USA; Cat#11965092) supplemented with 10% fetal bovine serum (FBS; Gibco, Grand Island, NY, USA; Cat#A5670701) and 1% penicillin-streptomycin (Gibco, Grand Island, NY, USA; Cat#15140122) at 37°C in 5% CO_2_.

### 2.9 Cell Treatment

HSC-3 and OSC-20 cells were seeded in 24-well plates at 5 × 10^4^ cells per well. Cells were treated with the bromodomain-containing domain 4 (BRD4) inhibitor JQ1 (Sigma-Aldrich, St. Louis, MO, USA; Cat#SML0974) at 0, 200, or 500 nM for 24 h. For DHFR inhibition, cells were treated with 0, 0.75, 1.5, or 3.0 μM methotrexate (MTX; Selleck Chemicals, Houston, TX, USA; Cat#S1210) for 24 h.

### 2.10 Quantitative Reverse Transcription-PCR (qRT-PCR)

Total RNA was extracted from HSC-2, HSC-3, and OSC-20 cells using TRIzol reagent (Invitrogen, Carlsbad, CA, USA; Cat#15596026CN). mRNA was reverse-transcribed to cDNA using the PrimeScript RT reagent Kit with gDNA Eraser (TaKaRa, Beijing, China; Cat#RR047A). Quantitative amplification was performed on a 7500 Real-Time PCR System (Applied Biosystems, Foster City, CA, USA) using the PowerTrack SYBR Green Master Mix (ThermoFisher Scientific, Waltham, MA, USA; Cat#A46110) with 20 ng cDNA per reaction. Relative gene expression was quantified using the 2^−ΔΔCt^ method with GAPDH as the internal reference. The primer sequences used for qRT-PCR are as follows:

DHFR: forward, 5^′^-CGCGAGCACGCCGCGACCCTGCGT-3^′^; reverse, 5^′^-CGCCCCCCTCGTCCC CATT-3^′^.

ELK4: forward, 5^′^-AGTGGGCAGGATTGAGGGT-3^′^; reverse, 5^′^-GCCAGTTTCTCGGCTGGATT-3^′^.

E2F1: forward, 5^′^-ACGCTATGAGACCTCACTGAA-3^′^; reverse, 5^′^-TCCTGGGTCAACCCCTCAAG-3^′^.

GAPDH: forward, 5^′^-GGAGCGAGATCCCTCCAAAAT-3^′^; reverse, 5^′^-GGCTGTTGTCATACTTC TCATGG-3^′^.

### 2.11 Chromatin Immunoprecipitation Followed by Quantitative PCR (ChIP-qPCR)

Cellular chromatin was cross-linked with 1% formaldehyde (Beyotime, Shanghai, China; Cat#P0099) for 15 min and quenched with 0.125 M glycine (Beyotime, Shanghai, China; Cat#ST085) for 5 min. HSC-3 and OSC-20 cells were harvested and lysed in ChIP-lysis buffer (Millipore, Billerica, MA, USA; Cat#20-163). Lysates were sonicated using a QSonica Q700 sonicator (Qsonica, Newtown, CT, USA) to shear chromatin DNA to approximately 200 bp. Lysates were incubated with anti-H3K27ac (1:500 dilution; Abcam, Cambridge, MA, USA; Cat#ab4729) and anti-ELK4 (1:100 dilution; Santa Cruz Biotechnology, Santa Cruz, CA, USA; Cat#sc-166823) at 4°C overnight. Antibody-chromatin complexes were incubated with Pierce protein A/G magnetic beads (ThermoFisher Scientific, Waltham, MA, USA; Cat#88803) for 2 h. The immunoprecipitated chromatin was eluted using elution buffer and then treated with 50 µg/mL RNase A (Beyotime, Shanghai, China; Cat#ST577) and 200 µg/mL proteinase K (Beyotime, Shanghai, China; Cat#ST535). DNA was purified using phenol/chloroform/isoamyl alcohol extraction and analyzed by qPCR. The primer sequences used for ChIP-qPCR are as follows:

E1: forward, 5^′^-CCAACTACTGCCTCCACAGG-3^′^; reverse, 5^′^-CTCCCCTATCCCTTTCCCCA-3^′^.

E2: forward, 5^′^-GTTGTTACCTTTGGGAATGGGGA-3^′^; reverse, 5^′^-CCACCACGACCAAC AAAGTAGT-3^′^.

E3: forward, 5^′^-CGACGATGCAGTTTAGCGAAC-3^′^; reverse, 5^′^-GCAGGCTTCCGGCGAG-3^′^.

### 2.12 Cell Transfection

Lentiviral vectors containing the full-length coding sequence of DHFR and corresponding empty vectors were purchased from Genepharma (Shanghai, China). For lentiviral packaging, 293T cells were transfected with lentiviral vectors using Lipofectamine 3000 (Invitrogen, Carlsbad, CA, USA; Cat#L3000150). Viral supernatants were collected 72 h post-transfection and used to infect HSC-3 and OSC-20 cells.

siRNAs targeting ELK4 (si-ELK4), E2F1 (si-E2F1), DHFR (si-DHFR), and non-targeting negative control siRNA (si-NC) were purchased from Genepharma (Shanghai, China). siRNAs were transfected using Lipofectamine 3000 (Invitrogen, Carlsbad, CA, USA; Cat#L3000150) according to the manufacturer’s instructions. Briefly, 50 nM siRNA was mixed with 5 μL serum-free DMEM containing 0.1 μL Lipofectamine 3000, followed by incubation at room temperature for 20 min. Then, 100 μL cell suspension (5 × 10^3^ cells per well) was added to 6-well plates pre-loaded with the transfection mixture and incubated at 37°C for 48 h.

### 2.13 Western Blot

HSC-3 and OSC-20 cells were lysed in radioimmunoprecipitation assay (RIPA) buffer (Millipore, Billerica, MA, USA; Cat#20-188). Protein concentration was measured using a BCA Protein Assay Kit (Beyotime, Shanghai, China; Cat#P0012). Lysates were mixed with loading buffer, denatured at 95°C for 5 min, and separated by 10% SDS-PAGE. Proteins were transferred onto polyvinylidene fluoride (PVDF) membranes (Millipore, Billerica, MA, USA; Cat#HVLP02500). Membranes were blocked with 5% skim milk for 2 h at room temperature, and incubated with primary antibodies at 4°C overnight. After washing with TBST buffer, membranes were incubated with Horseradish peroxidase (HRP)-linked goat anti-rabbit secondary antibody (1:10,000 dilution; Abcam, Cambridge, MA, USA; Cat#ab205718) for 1 h at room temperature. GAPDH was used as the internal reference. Protein bands were detected using the enhanced chemiluminescence (ECL) reagent (Beyotime, Shanghai, China; Cat#P0018HS) and analyzed by ImageJ software (version 1.52). Primary antibodies as follows: anti-ELK4 (1:5000 dilution; Santa Cruz Biotechnology, Santa Cruz, CA, USA; Cat#sc-166823), anti-E2F1 (1:5000 dilution; Abcam, Cambridge, MA, USA; Cat#ab314311), anti-DHFR (1:5000 dilution; Abcam, Cambridge, MA, USA; Cat#ab288373) and anti-GAPDH (1:5000 dilution; Abcam, Cambridge, MA, USA; Cat#ab181602). The original Western blot images were shown in Supplementary Fig. S1.

### 2.14 Matrigel-Based Tube Formation Assay

Pre-chilled 96-well plates were coated with 50 μL Matrigel (Corning, NY, USA; Cat#356235) and incubated at 37°C for 30 min to gelatinize. HSC-3 and OSC-20 cells (suspended in serum-free DMEM) were seeded into 96-well plates at 2 × 10^4^ cells per well and incubated at 37°C for 12 h. Cells were imaged using an inverted microscope (Olympus IX73; Olympus, Tokyo, Japan) at 200× magnification.

### 2.15 Cell Invasion Assay

Upper chambers of 24-well Transwell plates (Corning, NY, USA; Cat#CLS3421) were coated with Matrigel (Corning, NY, USA; Cat#356235). HSC-3 and OSC-20 cells suspended in serum-free DMEM were seeded into the upper chamber at 3 × 10^5^ cells per well in 200 μL. The lower chamber contained 600 μL DMEM with 10% FBS. After incubation for 24 h at 37°C, non-invading cells on the upper membrane surface were removed. Invaded cells on the lower membrane surface were fixed with 4% paraformaldehyde (Beyotime, Shanghai, China; Cat#P0099) and stained with 0.1% crystal violet (Beyotime, Shanghai, China; Cat#Y268090). Stained cells were photographed and counted under an inverted microscope (Olympus IX73; Olympus, Tokyo, Japan) at 200× magnification.

### 2.16 Statistical Analysis

Data are presented as mean ± standard deviation (SD) from at least three independent experiments. Statistical analysis was performed using R (version 4.4.3) or GraphPad Prism (version 10.4.0; GraphPad Software Inc., San Diego, CA, USA). Differences between the two groups were assessed by Student’s *t*-test. Comparisons among multiple groups were analyzed by one-way analysis of variance (ANOVA) with Tukey’s post hoc test. Spearman’s correlation coefficients were computed along with the 95% confidence intervals (CIs) using a bias-corrected and accelerated bootstrap method with 1000 resamples. *p* < 0.05 was considered significant.

## 3 Results

### 3.1 Expression of VM-Related Genes Correlated with Poor Prognosis in OSCC Patients

**Figure 1:**
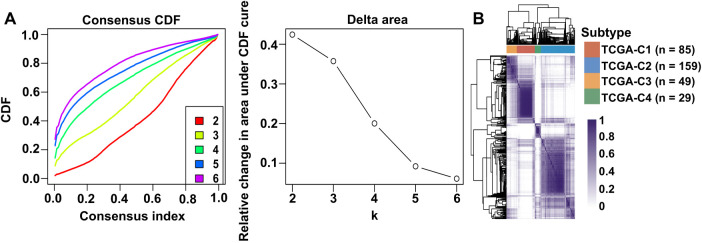

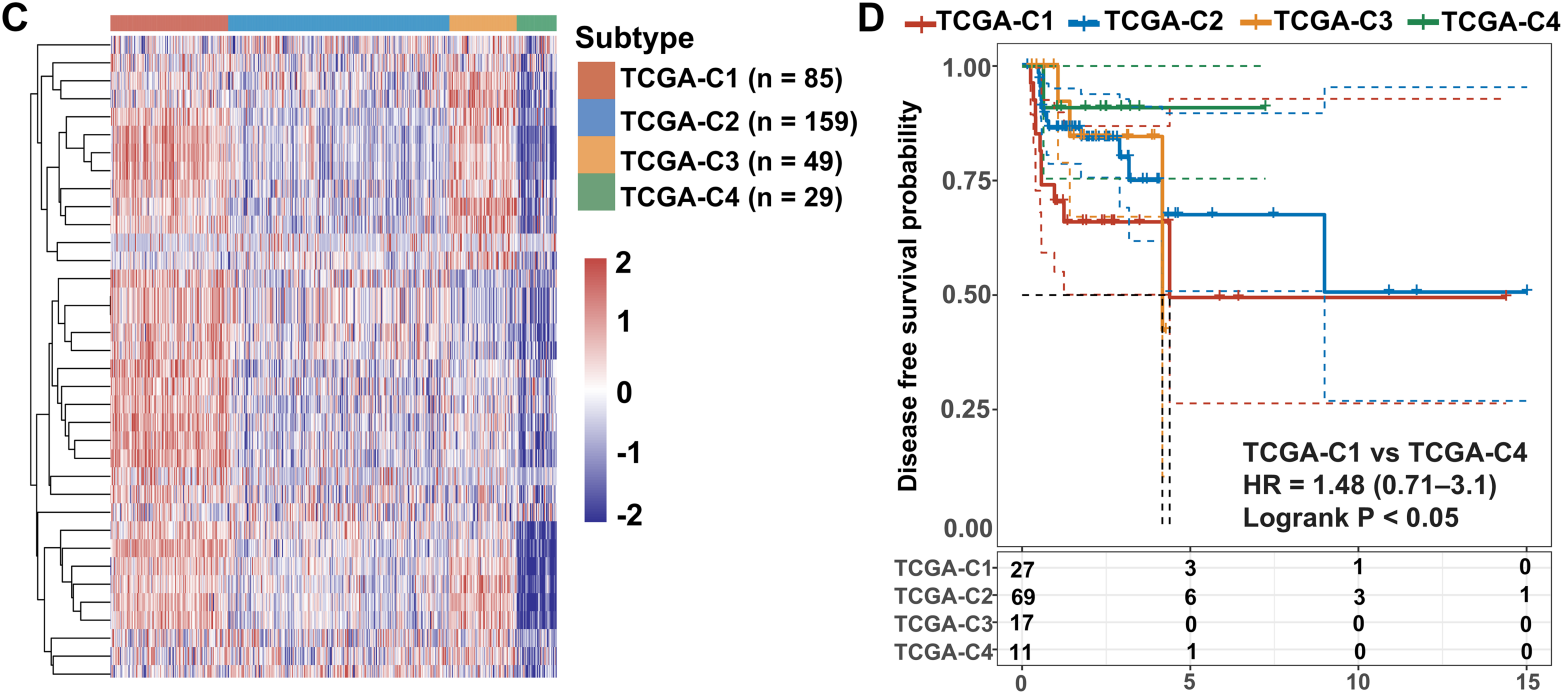
Expression of VM-related genes correlated with poor prognosis in OSCC patients. Bulk RNA-seq and clinical data from OSCC patients were obtained from the TCGA-OSCC cohort. (**A**) Cumulative distribution function (CDF) curves and derived delta area curves revealed k = 4 as the optimal cluster number for sample stratification. (**B**) Consensus matrix heatmap (k = 4) classified 322 OSCC samples into four molecular subtypes, including TCGA-C1 (*n* = 85), TCGA-C2 (*n* = 159), TCGA-C3 (*n* = 49), and TCGA-C4 (*n* = 29). (**C**) The heatmap showed VM-related gene expression across four molecular subtypes. (**D**) Disease-free survival analysis revealed significantly worse prognosis in TCGA-C1 vs. TCGA-C4 subtypes

Unsupervised clustering of VM-related genes in the TCGA-OSCC cohort was performed to stratify samples into distinct molecular subtypes. The optimal number of clusters was determined by the CDF curves, which yielded an optimal division at k = 4 (Fig. 1A). This analysis revealed four molecular subtypes, designated TCGA-C1, TCGA-C2, TCGA-C3, and TCGA-C4 (Fig. 1B). Notably, the TCGA-C1 subtype displayed the highest expression levels of VM-related genes, while the TCGA-C4 subtype exhibited the lowest (Fig. 1C). Kaplan-Meier survival analysis revealed that patients with the TCGA-C4 subtype exhibited significantly better outcomes compared to those classified as TCGA-C1 (Fig. 1D). These findings indicated that elevated VM-related gene expression correlated with adverse prognosis in OSCC patients.

### 3.2 scRNA-Seq Analysis Revealed Cellular Heterogeneity within the OSCC Microenvironment

**Figure 2:**
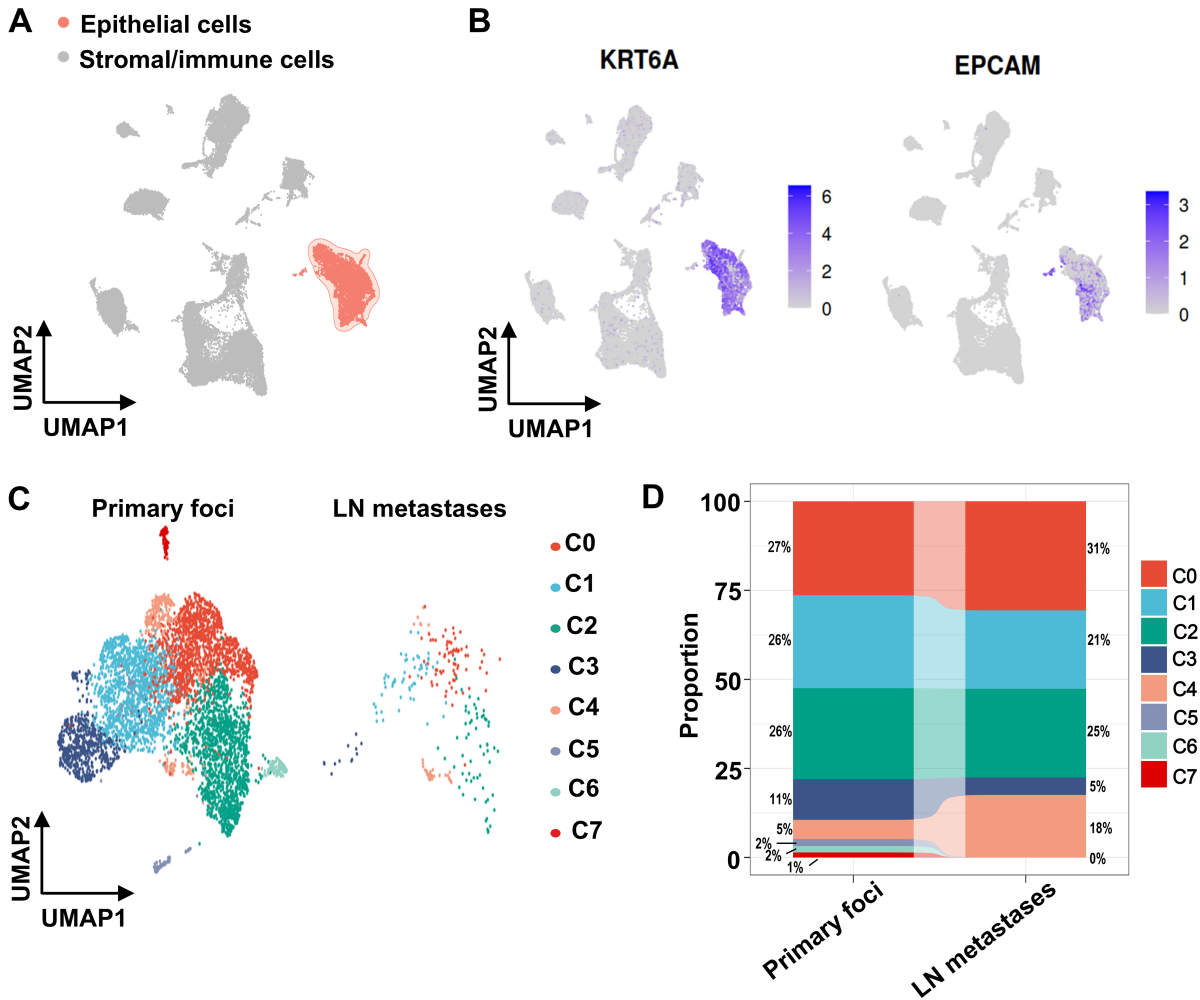
scRNA-seq analysis using the GSE181919 dataset revealed cellular heterogeneity within the OSCC microenvironment. (**A**) UMAP nonlinear dimensionality reduction identifying two major cell clusters (epithelial cluster and stromal/immune cell cluster) derived from primary OSCC foci and lymph node metastases. (**B**) Expression distribution of canonical epithelial marker genes (KRT6A and EPCAM) in primary OSCC foci and lymph node metastases. (**C**) Epithelial subcluster composition in primary OSCC foci and lymph node metastases. (**D**) Cell proportions of epithelial subclusters in primary OSCC foci and lymph node metastases. LN, lymph node

To elucidate mechanisms underlying VM and metastasis in OSCC, we analyzed single-cell transcriptomes from OSCC tissues using the GSE181919 dataset. UMAP dimensionality reduction classified cells from primary foci and lymph node metastases into two major clusters: an epithelial cluster and a stromal/immune cell cluster (Fig. 2A). Given the epithelial origin of OSCC [1], the epithelial cluster was confirmed by expression of epithelial cell marker genes, keratin 6A (KRT6A) and epithelial cell adhesion molecule (EPCAM), which distinguished epithelial cells from other cell types (Fig. 2B). Subsequently, we categorized the epithelial cluster into subclusters separately for primary foci and lymph node metastases (Fig. 2C). Primary foci contained eight epithelial subclusters, while lymph node metastases comprised five subclusters (Fig. 2C). Lymph node metastases exhibited higher proportions of subclusters C0 (31% vs. 27% in primary foci) and C4 (18% vs. 5% in primary foci), with subcluster C4 showing the most pronounced elevation (Fig. 2D). In contrast, subclusters C5, C6, and C7 were exclusively present in primary foci, absent from lymph node metastases (Fig. 2D). Collectively, these findings identified epithelial subclusters in OSCC and revealed enrichment of subclusters C0 and C4 in lymph node metastases.

### 3.3 hdWGCNA Identified Key Co-Expression Modules Associated with VM and Metastasis in OSCC

**Figure 3:**
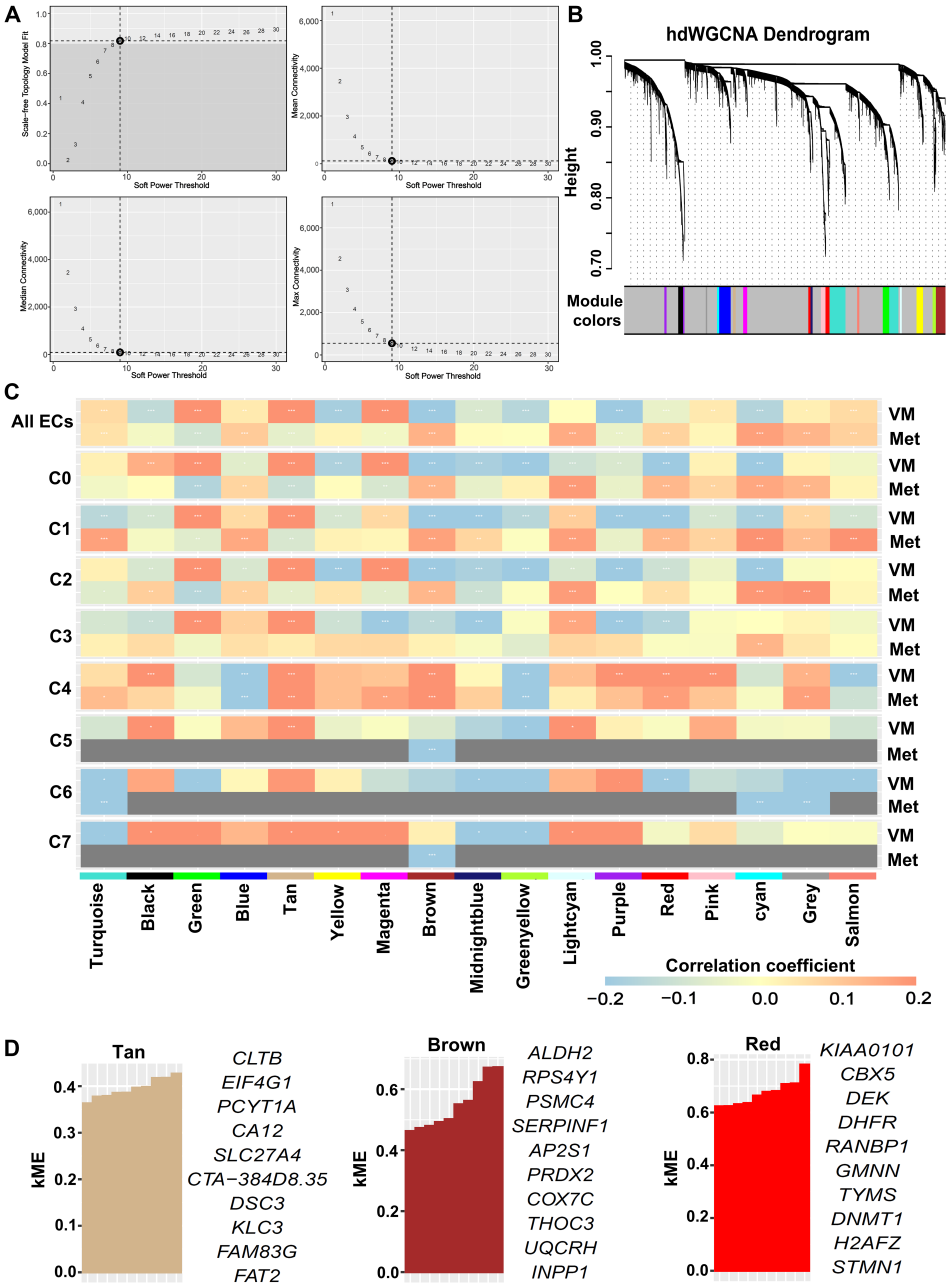
hdWGCNA identified key co-expression modules associated with VM and metastasis in OSCC. scRNA-seq data were derived from epithelial cells in the GSE181919 dataset. (**A**) Optimal soft threshold was set to 9, achieving maximal scale-free topology model fit and minimal connectivity. (**B**) Dendrogram of hierarchical clustering identifying 17 co-expression modules (including 16 non-gray modules) at soft threshold 9. (**C**) Heatmap of Spearman’s correlations between co-expression modules and VM/metastasis across all epithelial cells and subclusters. *False discovery rate (FDR)-adjusted *p* < 0.05; **FDR-adjusted *p* < 0.01; ***FDR-adjusted *p* < 0.001. ECs, epithelial cells; VM, vasculogenic mimicry; Met, metastasis. (**D**) Top 10 eigengenes from the tan, brown, and red modules ranked by kME values

To investigate transcriptional regulatory networks associated with VM and metastasis in OSCC, we applied hdWGCNA to scRNA-seq data from the epithelial cluster in the GSE181919 dataset. A scale-free co-expression network was constructed at the soft threshold of 9 (Fig. 3A). Hierarchical clustering revealed 17 modules, including 16 non-gray modules (Fig. 3B). To systematically evaluate the relationship between gene co-expression modules and malignant phenotypes, we generated a correlation heatmap analyzing associations with both VM and metastasis across all epithelial subclusters. Strikingly, epithelial subcluster C4 exhibited a unique correlation pattern, with three modules—tan, brown, and red—demonstrating strong positive correlations with both VM and metastasis compared to the same modules in other epithelial subclusters (Fig. 3C). Then, we calculated the kME values of eigengenes within the three modules. The top 10 eigengenes for each module were ranked according to kME values (Fig. 3D). Collectively, these findings suggested that the tan, brown, and red modules critically regulate VM and metastasis in OSCC, with their 30 eigengenes representing candidate mediators of these processes.

### 3.4 DHFR Was Identified as a Critical Regulator of VM and Metastasis in OSCC

**Figure 4:**
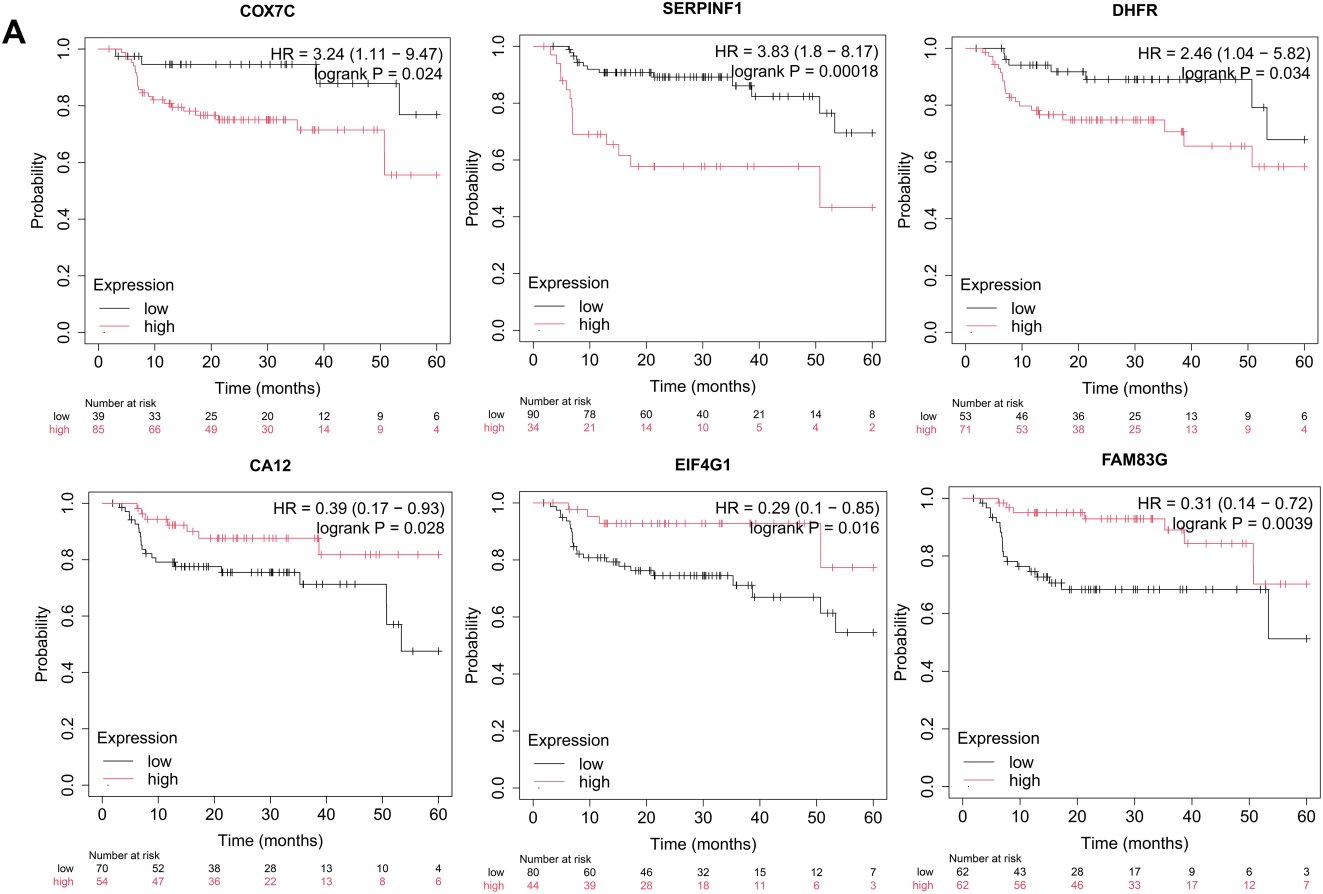

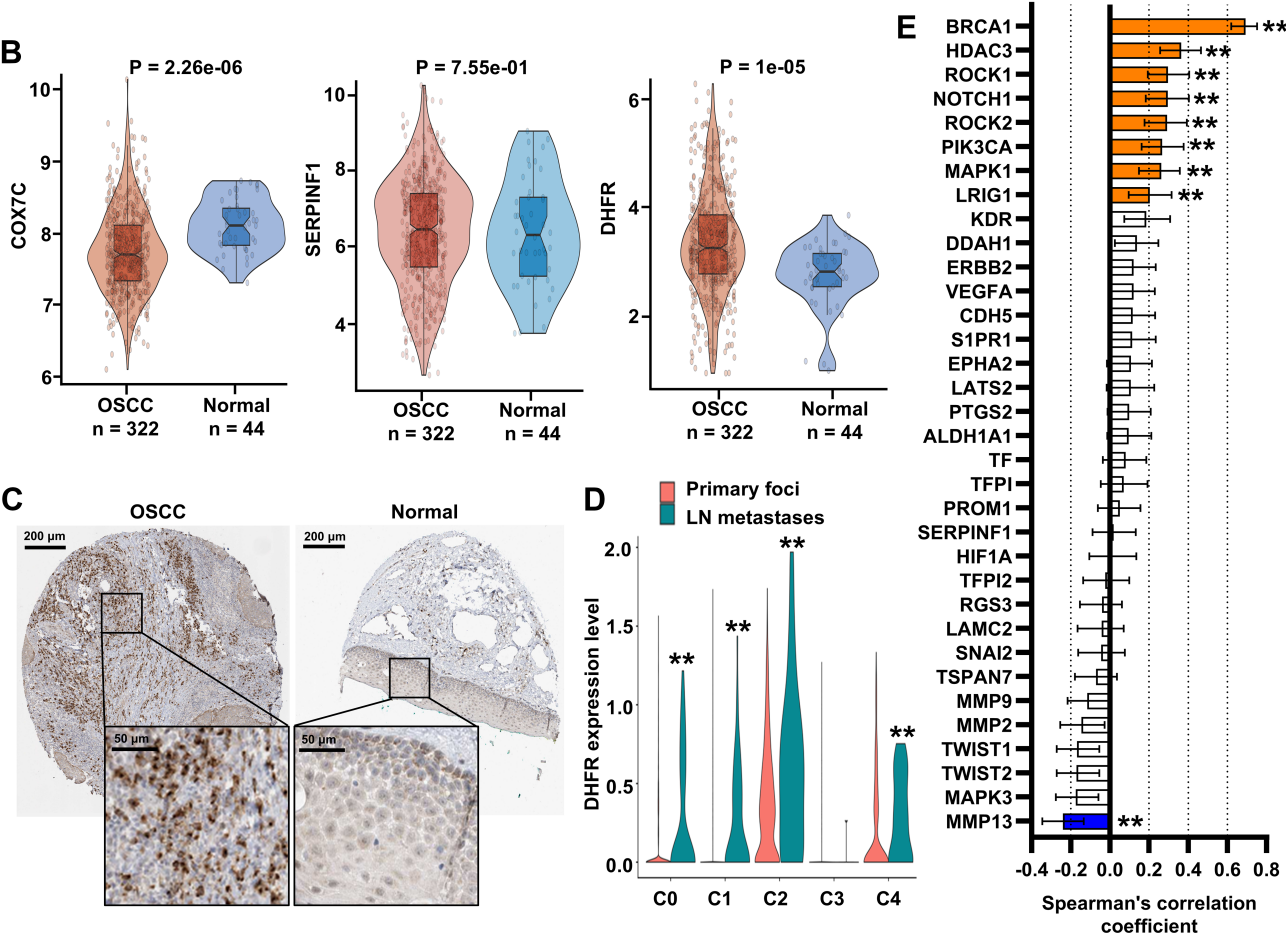
DHFR was identified as a critical regulator of VM and metastasis in OSCC. (**A**) Kaplan-Meier analysis indicated that the expression levels of COX7C, SERPINF1, DHFR, CA12, EIF4G1, and FAM83G were significantly associated with recurrence-free survival (RFS) in OSCC patients. Kaplan-Meier survival analysis was performed using the Kaplan-Meier Plotter platform (https://kmplot.com/analysis/). Patients were stratified into high- and low-expression groups using an automatically determined optimal cutoff value. Log-rank test was used to compare survival between high- and low-expression groups. (**B**) Differential expression of COX7C, SERPINF1, and DHFR between OSCC (*n* = 322) and normal control tissues (*n* = 44). Bulk RNA-seq data were obtained from the TCGA-OSCC cohort. Student’s *t*-test was applied to compare two groups. Data are shown as medians and interquartile ranges. (**C**) Representative immunohistochemistry images of DHFR in OSCC and normal control tissues were obtained from the Human Protein Atlas database (https://www.proteinatlas.org/). (**D**) Expression levels of DHFR across epithelial subclusters C0–C4 derived from lymph node metastases vs. primary foci. scRNA-seq data were obtained from the GSE181919 dataset. LN, lymph node. **p < 0.01 (Student’s *t*-test). (**E**) Spearman’s correlations between DHFR and VM marker genes in OSCC tissues. Bulk RNA-seq data were obtained from the TCGA-OSCC cohort. Data represent mean ± 95% confidence intervals (CIs). **FDR-adjusted *p* < 0.01

To identify key drivers of VM and metastasis in OSCC, we evaluated the prognostic implications of 30 eigengenes screened by hdWGCNA. Kaplan-Meier survival analysis showed six genes significantly associated with RFS in OSCC (Fig. 4A). High expression of COX7C, SERPINF1, and DHFR correlated with poor prognosis, whereas elevated CA12, EIF4G1, and FAM83G expression was associated with favorable outcomes (Fig. 4A). Then, we analyzed the expression of COX7C, SERPINF1, and DHFR in OSCC and normal control tissues using the TCGA-OSCC cohort. Compared to normal control tissues, DHFR was significantly upregulated in OSCC tissues, while COX7C showed significant downregulation and SERPINF1 exhibited no differential expression (Fig. 4B). Immunohistochemistry data from the Human Protein Atlas database confirmed that the protein levels of DHFR were obviously higher in OSCC than in normal control tissues (Fig. 4C). At single-cell resolution, DHFR was significantly upregulated in epithelial subclusters C0, C1, C2, and C4 of lymph node metastases compared to primary foci (Fig. 4D). Furthermore, we analyzed the expression correlations between DHFR and VM marker genes using the TCGA-OSCC cohort, with thresholds of *p* < 0.01 and |r| > 0.2 for significant correlation. DHFR expression was significantly positively correlated with eight VM markers and negatively correlated with MMP13 (Fig. 4E). Collectively, these findings defined DHFR as a critical regulator of VM and metastasis in OSCC.

### 3.5 Knockdown or Inhibition of DHFR Suppressed VM and Invasion in Metastatic OSCC Cells

**Figure 5:**
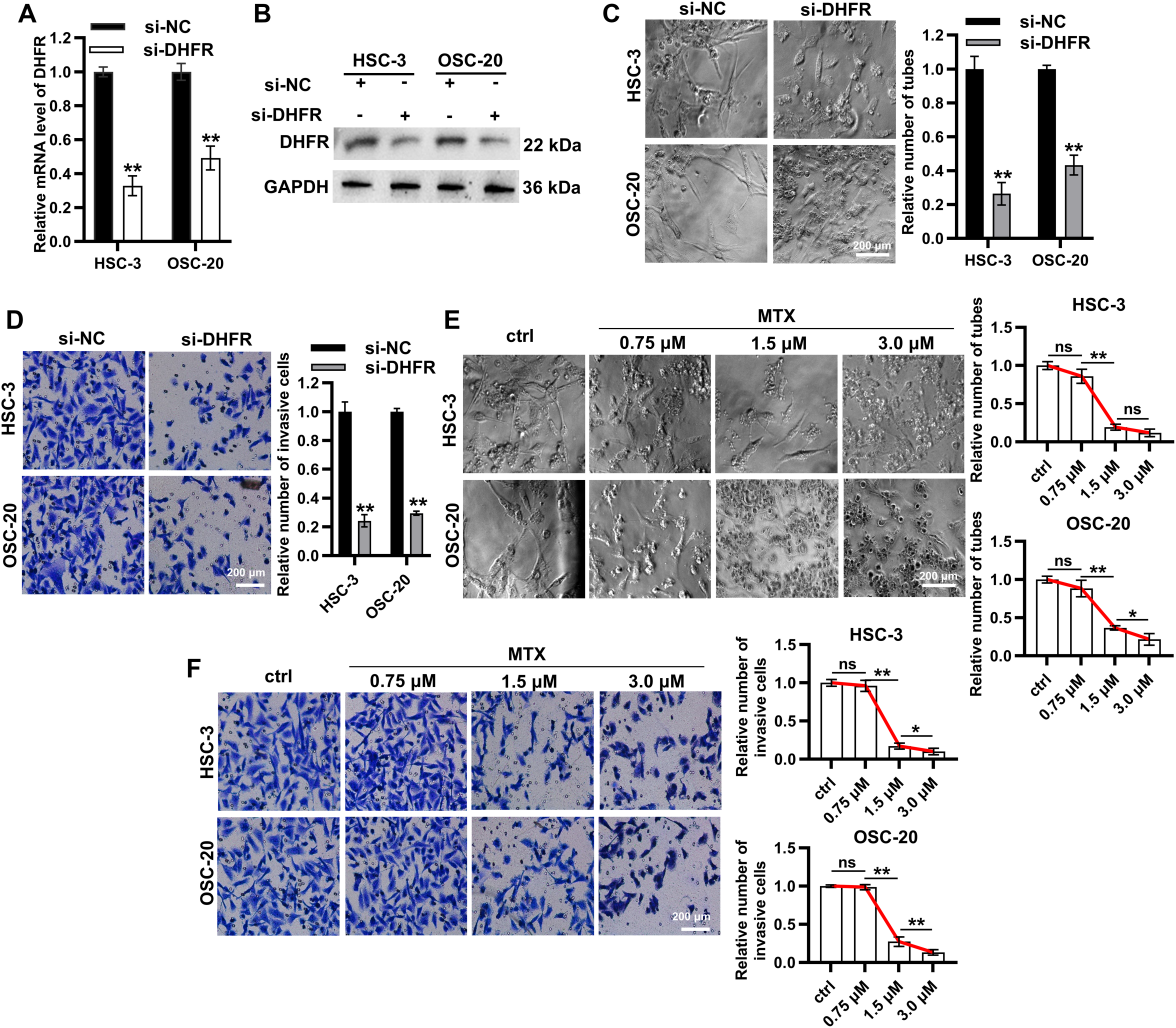
Knockdown or inhibition of DHFR suppressed VM and invasion in metastatic OSCC cells. (**A**,**B**) DHFR knockdown efficiency was confirmed using qRT-PCR (**A**) and Western blot (**B**) in HSC-3 and OSC-20 cells. Data are presented as mean ± SD. **p < 0.01 (Student’s *t*-test). (**C**,**D**) DHFR knockdown significantly inhibited VM tube formation ((**C**) 200× magnification) and invasion ((**D**): 200× magnification) in HSC-3 and OSC-20 cells. Data are presented as mean ± SD. **p < 0.01 (Student’s *t*-test). (**E**,**F**) Dose-dependent effects of MTX on VM tube formation ((**E**) 200× magnification) and invasive capacity ((**F**) 200× magnification) in HSC-3 and OSC-20 cells. MTX, methotrexate. Data are presented as mean ± SD. **p < 0.01; *p < 0.05; ns, non-significant (one-way ANOVA with Tukey’s post hoc test)

To investigate the functional role of DHFR, we generated DHFR-knockdown HSC-3 and OSC-20 cells (Fig. 5A,B). DHFR knockdown significantly reduced vascular-like tube formation in both HSC-3 and OSC-20 cells (Fig. 5C). Transwell assays demonstrated decreased invasion upon DHFR knockdown (Fig. 5D). To validate these findings, HSC-3 and OSC-20 cells were treated with gradient concentrations of MTX, an inhibitor of DHFR [23]. Compared with the control group, treatment with 0.75 μM MTX showed no significant effect on tube formation or invasion (Fig. 5E,F). In contrast, both 1.5 μM and 3.0 μM MTX significantly suppressed VM and invasive behavior in HSC-3 and OSC-20 cells (Fig. 5E,F). Notably, with the exception of tube formation in HSC-3 cells, where no significant difference was observed between 1.5 μM and 3.0 μM MTX, the higher concentration (3.0 μM) consistently resulted in a more pronounced suppression of both tube formation and cell invasion compared to 1.5 μM MTX (Fig. 5E,F). Collectively, these results indicated that knockdown or inhibition of DHFR suppressed VM and invasion in metastatic OSCC cells.

### 3.6 DHFR Was Identified as an Enhancer-Regulated Gene in Metastatic OSCC Cells

**Figure 6:**
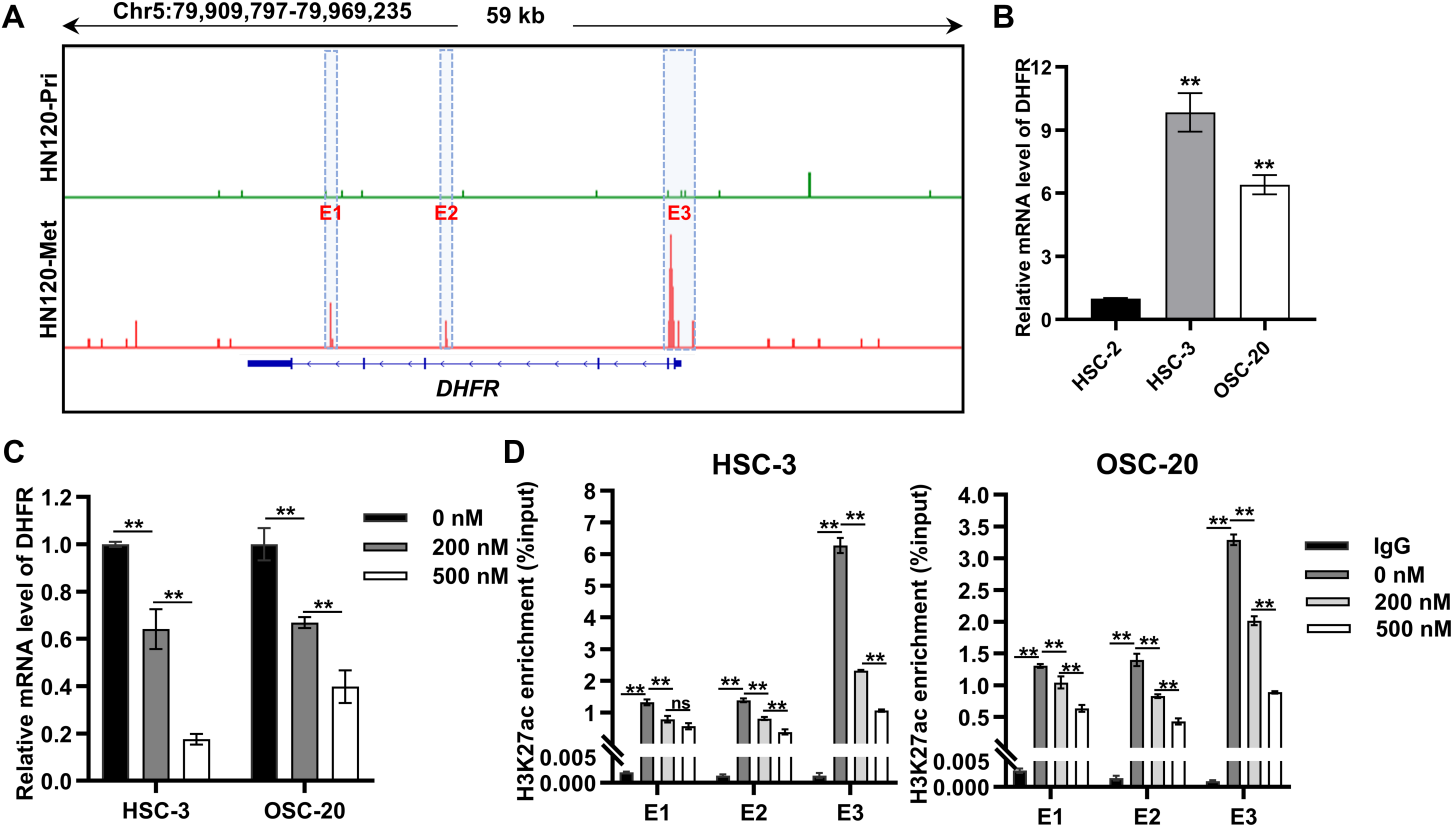
*DHFR* was identified as an enhancer-regulated gene in metastatic OSCC cells. (**A**) H3K27ac ChIP-seq profiles at the *DHFR* locus in primary (HN120-Pri) and metastatic (HN120-Met) OSCC cells. Data were obtained from the GSE120634 dataset. Three *DHFR* enhancer regions specific to HN120-Met cells were designated E1, E2, and E3. (**B**) DHFR mRNA levels in non-metastatic (HSC-2) and metastatic (HSC-3 and OSC-20) OSCC cells were detected by qRT-PCR. Data are presented as mean ± SD. **p < 0.01 vs. HSC-2 (one-way ANOVA with Tukey’s post hoc test). (**C**) JQ1-mediated DHFR transcriptional repression in HSC-3 and OSC-20 cells. Cells were treated with the indicated doses of JQ1 for 24 h. DHFR mRNA levels were detected by qRT-PCR. Data represent mean ± SD. **p < 0.01 (one-way ANOVA with Tukey’s post hoc test). (**D**) JQ1 treatment reduced H3K27ac enrichment at *DHFR* enhancer regions dose-dependently. Cells were treated with JQ1 for 24 h. H3K27ac enrichment at the three enhancer regions (E1, E2, and E3) was detected by ChIP-qPCR. Data represent mean ± SD. **p < 0.01; ns, non-significant (one-way ANOVA with Tukey’s post hoc test)

To elucidate the regulatory mechanisms governing DHFR expression, we analyzed H3K27ac enrichment at the *DHFR* locus in primary (HN120-Pri) and metastatic (HN120-Met) OSCC cells using ChIP-seq data from the GSE120634 dataset. Compared with HN120-Pri cells, HN120-Met cells displayed markedly increased H3K27ac signals at three enhancer regions (E1, E2, and E3) at the *DHFR* locus (Fig. 6A). Consistently, DHFR mRNA expression was significantly upregulated in metastatic OSCC cells (HSC-3 and OSC-20) relative to the non-metastatic OSCC cells (HSC-2) (Fig. 6B).

BRD4 binds acetylated enhancers and cooperates with transcription factors to activate target gene transcription [24–27]. To explore the regulation of DHFR expression, we employed JQ1, a BRD4 inhibitor that disrupts binding to acetylated lysine residues [28,29]. JQ1 induced dose-dependent suppression of DHFR mRNA expression in HSC-3 and OSC-20 cells (Fig. 6C). Consistently, JQ1 significantly reduced H3K27ac enrichment at *DHFR* enhancer regions dose-dependently, except at the E1 region in HSC-3 cells, where 200 nM and 500 nM JQ1 showed no significant difference (Fig. 6D).

Taken together, these results demonstrated *DHFR* was an enhancer-regulated gene in metastatic OSCC cells.

### 3.7 ELK4 Drove DHFR Transcription by Directly Binding to Its Enhancer Regions

**Figure 7:**
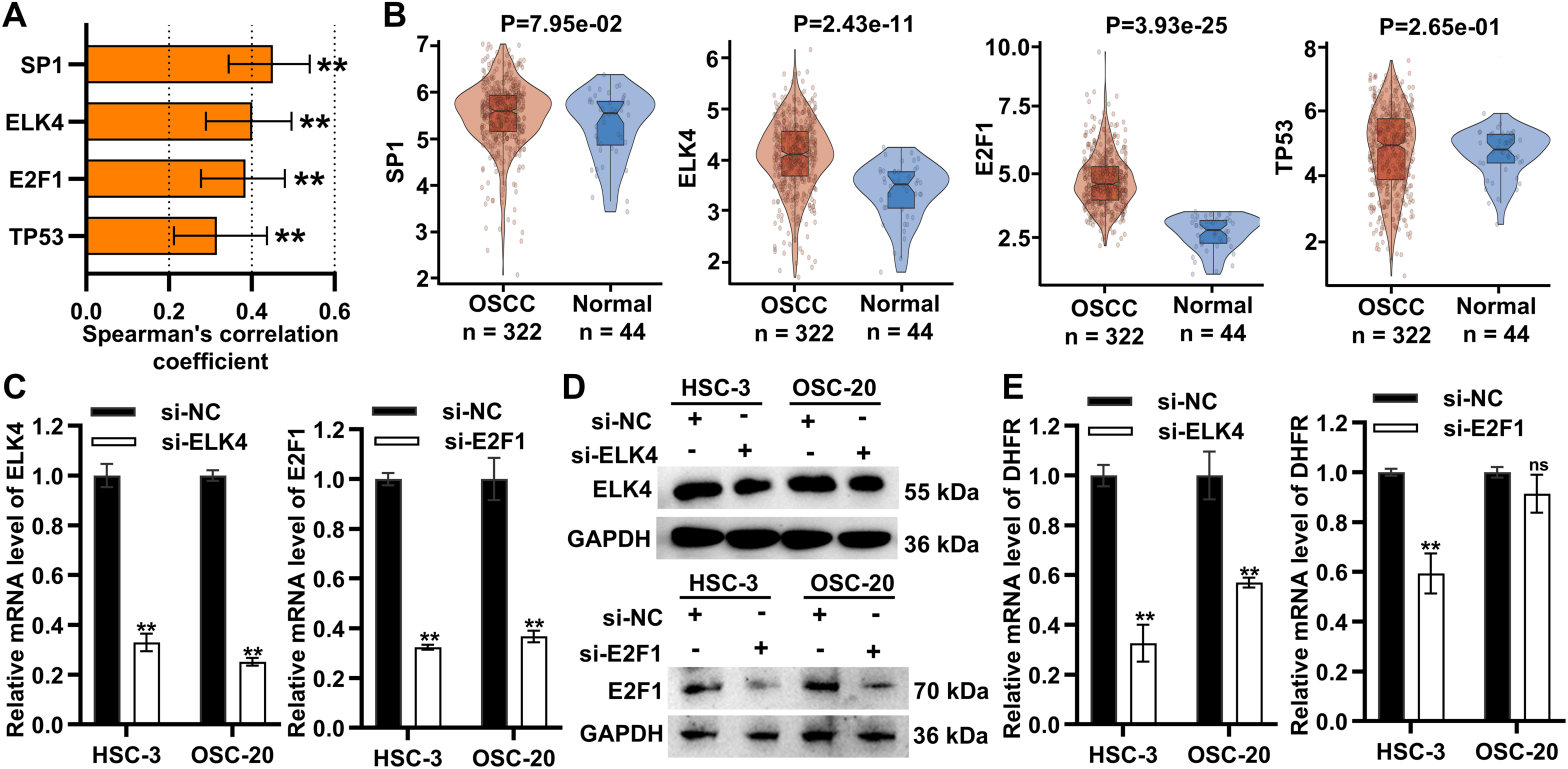

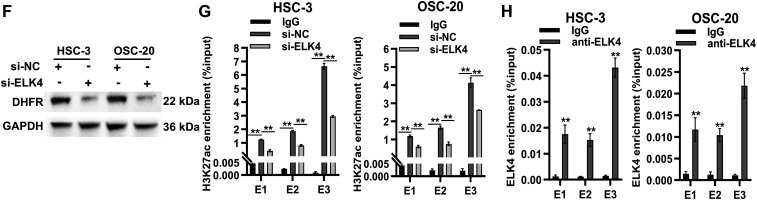
ELK4 drove *DHFR* transcription by directly binding to its enhancer regions. (**A**) Spearman’s correlations between transcription factors (SP1, ELK4, E2F1, and TP53) and DHFR expression in OSCC tissues. Data were derived from the TCGA-OSCC cohort. Data represent mean ± 95% CIs. **FDR-adjusted *p* < 0.01. (**B**) Differential expression of SP1, ELK4, E2F1, and TP53 in OSCC (*n* = 322) vs. normal control tissues (*n* = 44) based on the TCGA-OSCC cohort. Student’s *t*-test was applied to compare two groups. Data are shown as medians and interquartile ranges. (**C**,**D**) Knockdown efficiency of ELK4 and E2F1 in HSC-3 and OSC-20 cells confirmed using qRT-PCR (**C**) and Western blot (**D**). Data are presented as mean ± SD. **p < 0.01 (Student’s *t*-test). (**E**) Effects of ELK4 or E2F1 knockdown on DHFR mRNA levels in HSC-3 and OSC-20 cells were assessed by qRT-PCR. Data are presented as mean ± SD. **p < 0.01; ns, non-significant (Student’s *t*-test). (**F**) ELK4 knockdown reduced DHFR protein levels in HSC-3 and OSC-20 cells. (**G**) ELK4 knockdown inhibited H3K27ac enrichment at *DHFR* enhancer regions in HSC-3 and OSC-20 cells. H3K27ac enrichment was assessed by ChIP-qPCR. Data represent mean ± SD. **p < 0.01 (one-way ANOVA with Tukey’s post hoc test). (**H**) ChIP-qPCR confirmed direct binding of ELK4 to *DHFR* enhancer regions (E1–E3). Data represent mean ± SD. **p < 0.01 (Student’s *t*-test)

Transcription factors are central orchestrators of enhancer-mediated transcriptional activation. Among the top ten candidate transcription factors regulating *DHFR* (E2F1, TP53, HNF4A, SP1, PBX1, ELK4, Ar, Klf4, EBF1, and CEBPB), six (E2F1, TP53, HNF4A, SP1, PBX1, and ELK4) were identified as significant (Table 1). SP1, ELK4, E2F1, and TP53 showed significant positive correlations with DHFR expression (FDR-adjust *p* < 0.01; *r* > 0.2) (Fig. 7A). Compared to normal controls, OSCC tissues showed significantly elevated expression of ELK4 and E2F1, while SP1 and TP53 remained unchanged (Fig. 7B).

**Table 1:** Potential transcription factors for *DHFR*

Rank	Transcription factor	FDR adjust *p*-value
1	E2F1	0.002402
2	TP53	0.006301
3	HNF4A	0.01278
4	SP1	0.046538
5	PBX1	0.047963
6	ELK4	0.048565
7	Ar	0.053843
8	Klf4	0.057284
9	EBF1	0.065726
10	CEBPB	0.075491

To assess the functional roles of ELK4 and E2F1, we performed siRNA-mediated ELK4/E2F1 knockdown in HSC-3 and OSC-20 cells (Fig. 7C,D). ELK4 knockdown significantly suppressed DHFR transcription in both cell lines, while E2F1 knockdown inhibited DHFR transcription exclusively in HSC-3 cells (Fig. 7E). Additionally, Western blot demonstrated that ELK4 knockdown reduced DHFR protein levels in both HSC-3 and OSC-20 cells (Fig. 7F).

To examine whether ELK4 regulates *DHFR* enhancer activity, we assessed H3K27ac enrichment at the *DHFR* enhancer regions (E1–E3) upon ELK4 knockdown. ChIP-qPCR analysis revealed that ELK4 knockdown significantly reduced H3K27ac levels at these enhancer regions in both cell lines (Fig. 7G). Furthermore, we conducted ELK4 ChIP-qPCR to evaluate direct ELK4 occupancy at the E1–E3 regions. The results showed that ELK4 occupied the *DHFR* enhancer regions in both HSC-3 and OSC-20 cells (Fig. 7H).

Collectively, these results showed that ELK4 drove *DHFR* transcription by directly binding to its enhancer regions.

### 3.8 ELK4 Promoted DHFR Transcription to Facilitate VM and Invasion in Metastatic OSCC Cells

**Figure 8:**
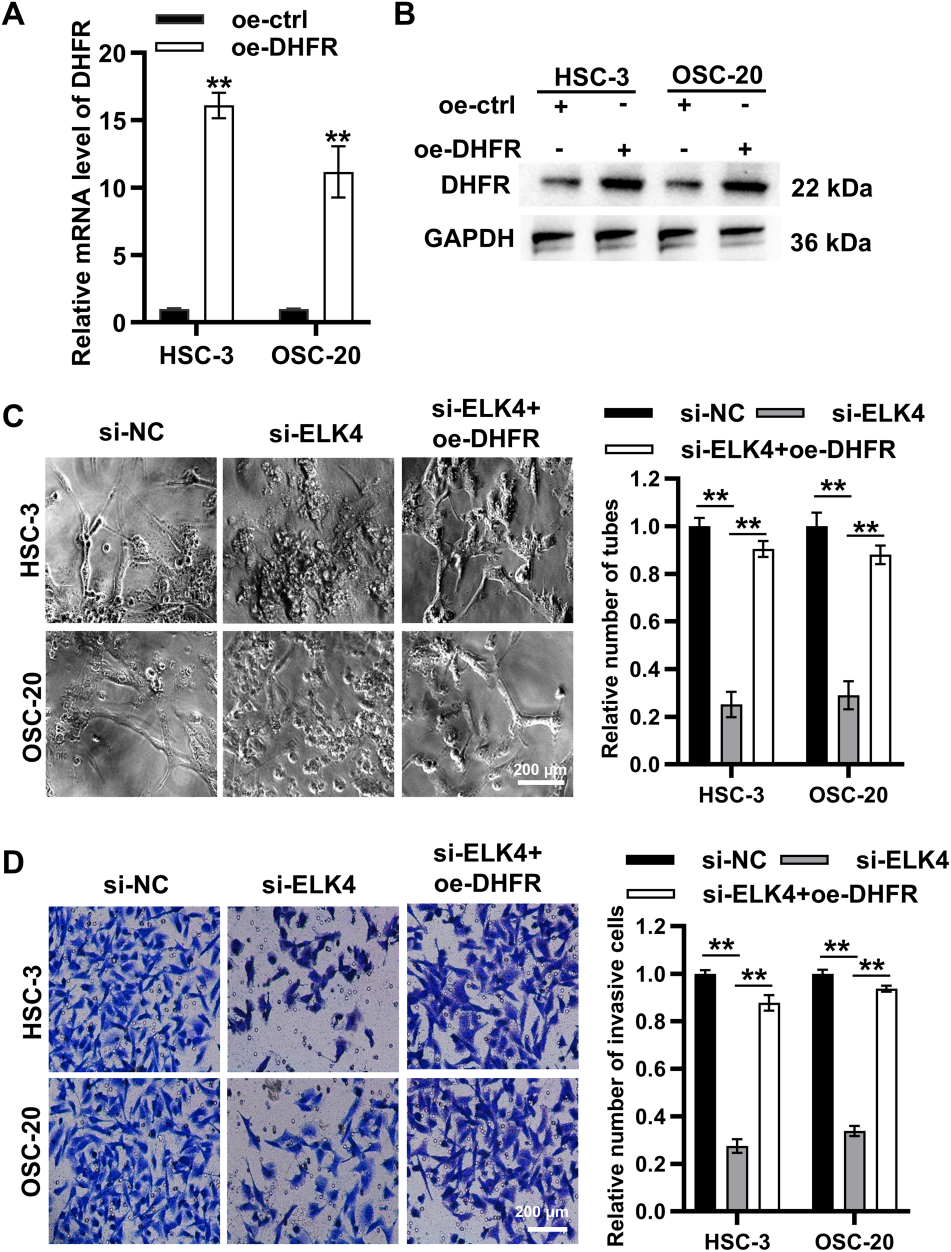
ELK4 promoted *DHFR* transcription to facilitate VM and invasion in metastatic OSCC cells. (**A**,**B**) DHFR overexpression efficiency in HSC-3 and OSC-20 cells was assessed by qRT-PCR (**A**) and Western blot (**B**). Data represent mean ± SD. **p < 0.01 (Student’s *t*-test). (**C**) ELK4 knockdown significantly suppressed VM tube formation, which was partially rescued by DHFR overexpression. Vascular-like tube formation in HSC-3 and OSC-20 cells was evaluated by Matrigel-based tube formation assays (200× magnification). Data represent mean ± SD. **p < 0.01 (one-way ANOVA with Tukey’s post hoc test). (**D**) ELK4 knockdown significantly inhibited invasion, a phenotype reversed by DHFR overexpression. Invasive capacity in HSC-3 and OSC-20 cells was assessed by Transwell assays (200× magnification). Data represent mean ± SD. **p < 0.01 (one-way ANOVA with Tukey’s post hoc test)

To explore the functional roles of the ELK4/DHFR axis, DHFR was overexpressed in HSC-3 and OSC-20 cells (Fig. 8A,B). ELK4 knockdown significantly inhibited vascular-like tube formation in both HSC-3 and OSC-20 cells, while DHFR overexpression partially rescued this inhibitory effect (Fig. 8C). Transwell assays indicated that ELK4 knockdown significantly reduced invasion in HSC-3 and OSC-20 cells, which was reversed by DHFR overexpression (Fig. 8D). Taken together, these results suggested that ELK4 promoted *DHFR* transcription to facilitate VM and invasion in metastatic OSCC cells.

## 4 Discussion

**Figure 9:**
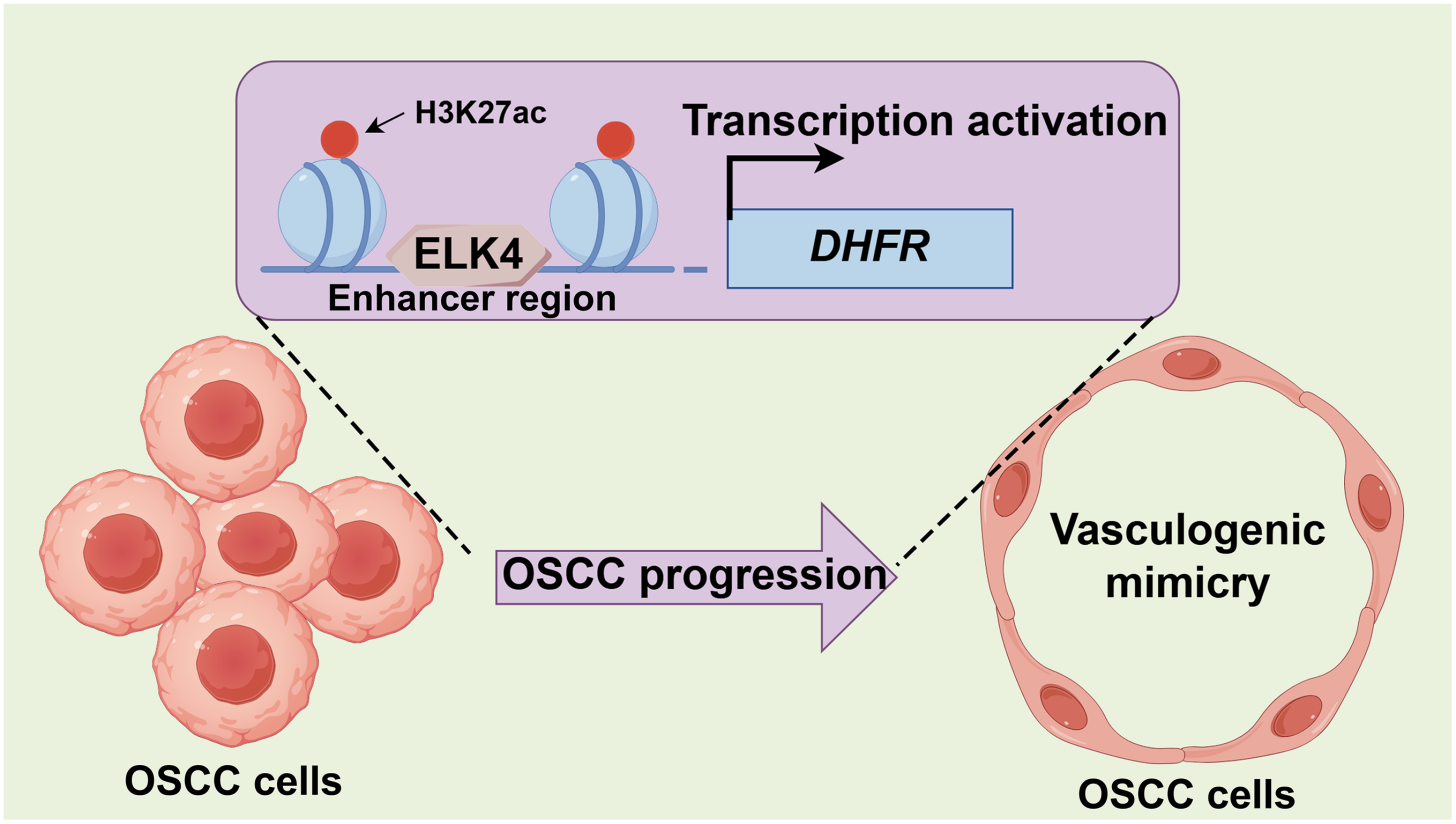
Proposed model for the ELK4/DHFR axis in promoting VM and metastasis in OSCC. ELK4 activates *DHFR* transcription through direct binding to its enhancer, thereby driving VM and metastatic progression in OSCC

Metastasis is a critical determinant of poor prognosis in OSCC [30,31]. Beyond conventional endothelium-dependent angiogenesis, VM represents an alternative vascularization mechanism that contributes to metastatic progression [4]. However, the regulatory mechanisms underlying VM in OSCC remain poorly understood. Using multi-omics approaches, we identified DHFR as a key mediator of VM and metastasis in OSCC. Mechanistically, ELK4 directly bound *DHFR* enhancer regions, increasing enhancer activity and activating *DHFR* transcription. Functionally, the ELK4/DHFR axis drove VM and invasion in OSCC (Fig. 9).

VM facilitates tumor growth through nutrient supply and metabolic waste removal [32]. Additionally, anastomosis of VM-derived vascular networks with endothelial vasculature enables systemic metastasis [33]. VM presence predicts poor overall survival in patients with head and neck squamous cell carcinoma [34]. In this study, we stratified OSCC samples from the TCGA database into four molecular subtypes, identifying the TCGA-C1 subtype with the highest expression of VM-related genes and worst prognosis. These results confirmed VM as an adverse prognostic factor in OSCC.

Regulatory mechanisms underlying VM in oral cancer are poorly defined. Previous studies have shown that IL-17F suppresses VM in oral tongue squamous cell carcinoma [35]; HIF-1α/EphA2/Laminin-5γ2 axis downregulation inhibits hypoxia-induced VM in oral cancer [36]; SOX7 inhibits VM by downregulating VE-cadherin in OSCC [37]. However, mechanisms governing VM plasticity in OSCC remain incompletely understood. In the present study, single-cell transcriptomic analysis revealed epithelial cell heterogeneity in OSCC tissues, with notable expansion of epithelial subclusters C0 and C4 (particularly C4) in lymph node metastases compared to primary foci. These findings suggested that subcluster C4 is a potential orchestrator of VM-mediated metastasis in OSCC.

To identify key regulators of VM and metastasis in OSCC, we applied hdWGCNA to epithelial single-cell transcriptomes. This analysis revealed that epithelial subcluster C4 was strongly associated with aggressive phenotypes, showing marked enrichment in lymph node metastases compared to primary foci, thus establishing subcluster C4 as a pivotal mediator of VM-related metastasis. Module–trait correlation analysis identified three co-expression modules positively associated with VM and metastatic potential. Prognostic analysis of module eigengenes identified three candidate drivers: COX7C, SERPINF1, and DHFR. Among these, only DHFR was upregulated in OSCC tissues compared to normal controls. Single-cell resolution analysis revealed that DHFR expression was significantly elevated in lymph node metastases across all epithelial subclusters except subcluster C3. Intriguingly, subcluster C3 displayed diminished abundance in lymph node metastases vs. primary foci.

DHFR catalyzes the reduction of dihydrofolate to tetrahydrofolate, serving as an essential enzyme in folate metabolism [38]. While DHFR inhibition with MTX is a well-established cancer treatment strategy [39], its pleiotropic functions in cancer progression remain unclear. DHFR modulates multiple oncogenic processes including proliferation, invasion, apoptosis, and stemness. For instance, DHFR promotes proliferation of breast cancer cells [40]. Inhibition of DHFR impairs self-renewal capacity while inducing differentiation in brain tumor-initiating cells [41]. In OSCC, disruption of DHFR substrate accessibility suppresses proliferation and invasion, while triggering apoptosis, autophagy, and cell cycle arrest [42]. Despite these findings, the potential role of DHFR in VM remains unexplored. Here, we demonstrated that knockdown or inhibition of DHFR significantly suppressed VM formation and invasive capacity in metastatic OSCC cells.

Enhancer-driven transcriptional reprogramming is a critical epigenetic mechanism for oncogene activation during malignant progression [14–16]. This study demonstrated that *DHFR* was an enhancer-regulated oncogene in metastatic OSCC cells, with ELK4 directly binding to specific enhancer regions to drive its transcription. ELK4, an Ets family transcription factor, exhibits diverse functions in cancer progression, typically acting as an oncogene that modulates processes such as angiogenesis, immune evasion, and metastasis. In colorectal cancer, hypoxia promotes the binding of phosphorylated PYCR1 to ELK4 and their recruitment to target gene promoters, accelerating tumor progression [43]; ELK4 cooperates with SP1/SP3 to activate LRG1 transcription, thereby enhancing tumor angiogenesis [44]; ELK4 persulfidation promotes an immunosuppressive microenvironment by upregulating AAK-1, which inhibits CD8^+^ T cell migration [45]; lncRNA SNHG16 facilitates immune evasion through the ELK4/PD-L1 axis [46]. In gastric cancer, ELK4 activates lncRNA SNHG22 transcription to inhibit tumor suppressor expression and upregulate the Notch1 signaling, facilitating cell proliferation and invasion [47]. ELK4-mediated Mcl-1 overexpression promotes oncogenesis in glioblastoma [48]. In HPV-positive cervical cancer, ELK4 promotes cell cycle progression and stemness by modulating the FBXO22/PTEN axis [49]. Conversely, ELK4 functions as a tumor suppressor in vestibular schwannoma by promoting HCG11 transcription, thereby inhibiting cell proliferation and inducing apoptosis [50]. Although ELK4 has been implicated as a potential regulator in head and neck squamous cell carcinoma [51], its function in OSCC remain unexplored. The present study demonstrated that ELK4 knockdown significantly inhibited VM formation and invasion in metastatic OSCC cells, effects rescued by DHFR overexpression. These results established the ELK4/DHFR axis as a critical driver of VM and invasion in OSCC.

## 5 Limitations of this Study

The following are the limitations of the current study. First, although we elucidated the role of the ELK4/DHFR axis in VM and metastasis *in vitro*, *in vivo* validation is required to establish its functional significance and therapeutic potential. Future investigations should assess the ELK4/DHFR axis *in vivo* and evaluate its potential as an anti-angiogenic target. Second, the precise mechanisms by which DHFR facilitates VM formation and metastasis remain poorly defined. Subsequent studies should investigate the downstream effectors of DHFR and their crosstalk with other signaling pathways.

## 6 Conclusion

This study identifies the ELK4/DHFR axis as a critical mediator of VM and metastasis in OSCC. We demonstrate that ELK4 activates *DHFR* transcription by directly binding to its enhancer regions, thereby facilitating VM formation and metastasis in OSCC. These findings establish a mechanistic foundation for VM regulation and position the ELK4/DHFR axis as a promising therapeutic target for complementing anti-angiogenic treatment in OSCC.

## Supplementary Materials



## Data Availability

The data that support the findings of this study are available from the corresponding author upon reasonable request.

## Supplementary Materials

The supplementary material is available online at https://www.techscience.com/doi/10.32604/or.2025.069612/s1.
