# Supplementary figures and images for: ELK4 Promotes Vasculogenic Mimicry in Oral Squamous Cell Carcinoma via Driving DHFR Transcriptional Activation
Source: Oncol Res. 2025 Dec 30;34(1):21. doi: 10.32604/or.2025.069612 (PMC12774561; doi:10.32604/or.2025.069612)

Supplementary Materials


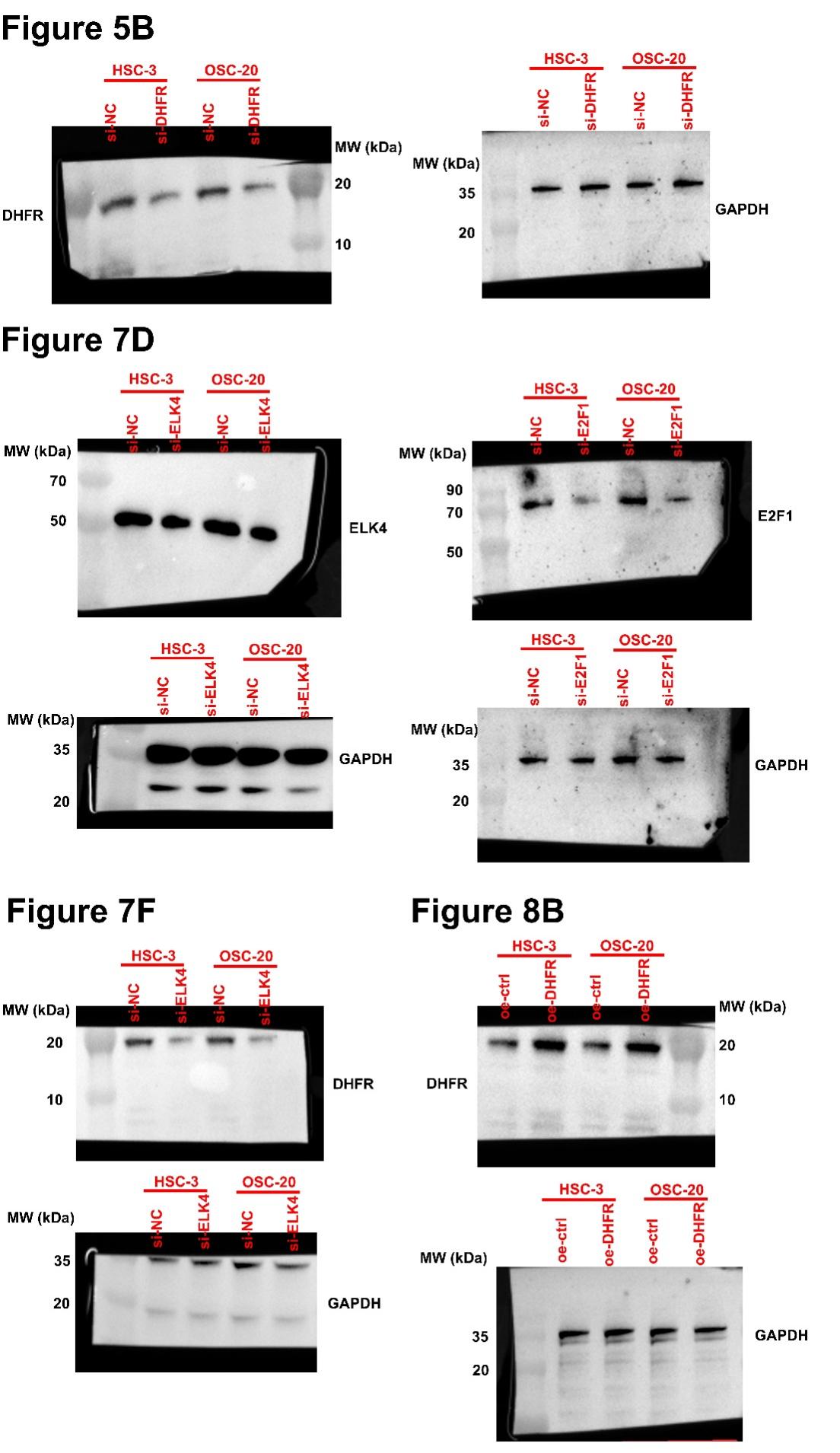


**Figure S1:** Original western blot images.

Supplement: Supplementary file 1 [file OncolRes-34-69612-s001.docx]
